# Development and psychometric evaluation of the Infertility Public Stigma Scale for Japanese women: protocol for an exploratory sequential mixed method study of the Japanese population

**DOI:** 10.3389/fpubh.2025.1504842

**Published:** 2025-05-21

**Authors:** Rie Yokota, Tsuyoshi Okuhara, Hiroko Okada, Emi Furukawa, Ritsuko Shirabe, Toshie Agawa, Hisashi Kato, Takahiro Kiuchi

**Affiliations:** ^1^Department of Medical Communication, School of Pharmacy and Pharmaceutical Sciences, Hoshi University, Tokyo, Japan; ^2^Department of Health Communication, School of Public Health, The University of Tokyo, Tokyo, Japan; ^3^University Hospital Medical Information Network (UMIN) Center, The University of Tokyo Hospital, Tokyo, Japan; ^4^School of Pharmacy and Pharmaceutical Sciences, Hoshi University, Tokyo, Japan; ^5^Chichibu Municipal Hospital, Saitama, Japan

**Keywords:** infertility, public stigma, scale development, exploratory sequential mixed method study, validity, reliability, psychometric evaluation, health communication

## Abstract

**Introduction:**

Women undergoing infertility treatment are often tagged with negative labels, subjected to negative reactions and behaviors by laypeople (public stigma), and they internalize these negative values (self-stigma). As self-stigma is associated with poor mental health, a measure is needed to determine the current state of public stigma in Japan and to evaluate the effectiveness of efforts to reduce it. However, existing instruments to measure public stigma in this context are limited. Therefore, this article aims to describe the research protocol for the development of the Infertility Public Stigma Scale for Japanese women and examination of its reliability and validity.

**Methods and analysis:**

This study will adopt an exploratory, sequential, mixed-methods design. In the qualitative research phase, the constructs and components of public stigma toward women undergoing infertility treatment will be explored based on interviews with Japanese laypeople. Eligible participants will be recruited through purposive sampling, ensuring maximum variation in sex, age, occupation, place of residence, medical history, and contact with women with primary or secondary infertility. Data will be analyzed using qualitative-descriptive methods and inductive thematic analysis to develop the initial scale. After examining the content validity through an expert panel and cognitive debriefing, laypeople will be surveyed online to test the scale’s validity and reliability. Quantitative research will be conducted using the initial scale. Structural validity will be examined using exploratory and confirmatory factor analyses. Known-groups validity will be tested based on the hypothesis that laypeople aged over 60 years exhibit higher levels of public stigma than younger individuals. Convergent validity will be tested under the hypothesis that individuals with higher levels of fertility knowledge will also report higher levels of public stigma. Convergent and discriminant validity will be examined using a multitrait-scaling analysis. Internal consistency will also be examined by calculating Cronbach’s alpha coefficients and item-total and item-remainder correlations.

**Discussion:**

The development of a reliable and validated public stigma scale for Japanese women undergoing infertility treatment will help understand the current state of public stigma. Simultaneously measuring the effectiveness of intervention studies to reduce public stigma toward women undergoing infertility treatment is also important.

## 1 Introduction

Infertility is the inability to get pregnant despite having unprotected intercourse for 1 year (1). Globally, 186 million people experience infertility (2). According to a survey conducted in Japan in 2021, 22.7% of couples (1 in 4.4) were undergoing or had undergone infertility testing or treatment (3). In Japan, infertility treatment has been covered by insurance since April 2022, and the number of women receiving such treatment is expected to increase.

Approximately half of the women undergoing infertility treatment have symptoms of anxiety, depression, or suspected anxiety or depression (4). Although there are many causes of psychological distress, a higher degree of self-stigma among women undergoing infertility treatment is associated with symptoms of anxiety or depression (4).

Infertility stigma refers to a series of processes in which (1) women undergoing infertility treatment are perceived as deviating from social expectations and are subjected to negative reactions and behaviors by the lay public (public stigma) and (2) they perceive these expectations, reactions, and behaviors (perceived stigma) and internalize these values (self-stigma) (5–7). Therefore, efforts to reduce self-stigma in women undergoing infertility treatment, as well as public stigma against them, are essential for maintaining their mental health (4, 8, 9). According to a Japanese survey in 2022, 20.2% of respondents stated that married couples were socially acceptable only after having a child (10).

Several scales have been developed and validated to measure self-stigma of women undergoing infertility treatment (8, 11, 12). In addition, public stigma scales have been developed for mental illnesses, cancer, HIV/AIDS, and other chronic diseases (13, 14). In the same fields, intervention programs to reduce public stigma have been implemented, including psychoeducation, educational materials, patient contact, and anti-stigma campaigns (videos and media) (13). However, no studies have examined the concept and components of public stigma toward women undergoing infertility treatment. In addition, no measures of public stigma toward this population exist in Japan or worldwide. Therefore, this research protocol describes a plan for the development of the Infertility Public Stigma Scale for Japanese women and the examination of its reliability and validity. This scale is expected to help understand the current state of public stigma toward women undergoing infertility treatment and measure the effectiveness of programs to reduce it.

## 2 Methods and analysis

**Figure 1:**
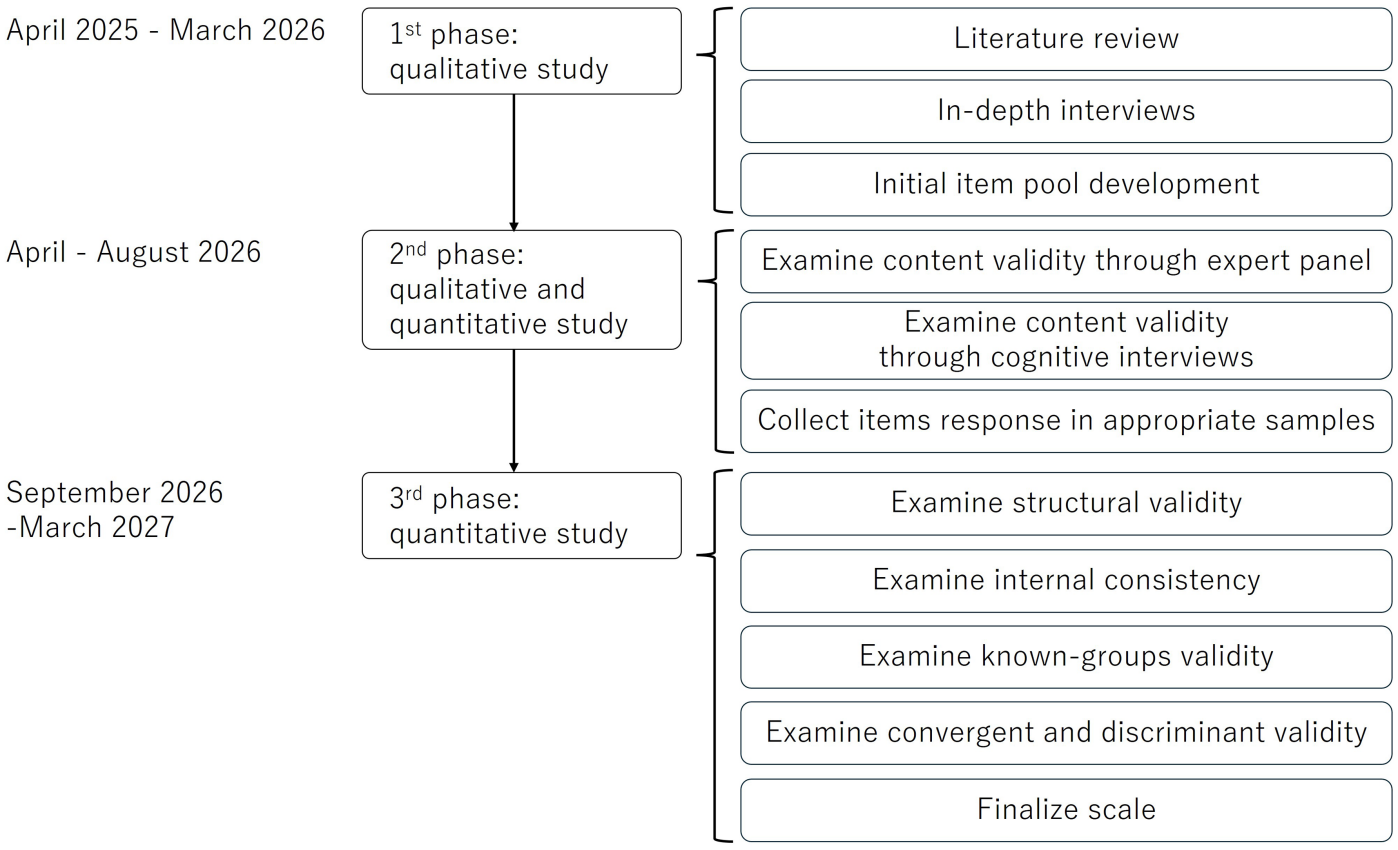
The scale development and evaluation process.

This is an exploratory, sequential, mixed-methods study (Figure 1). In this design, qualitative research is followed by quantitative research based on the hypotheses derived from the former (15). This design is suitable for scale development and validation (16). In the qualitative research phase, the constructs and components of public stigma toward women undergoing infertility treatment will be explored through interviews with the Japanese lay population, developing the initial items of the Infertility Public Stigma Scale for Japanese women. This phase is planned between April 2025 and March 2026 (Figure 1). After examining the content validity of the initial scale though expert panel discussions and cognitive interviews (April–August 2026), the psychometric properties of the scale will be examined quantitatively based on the initial items. This phase is expected to be completed by March 2027.

### 2.1 The qualitative phase: item generation

Multiple procedures are involved in item generation. First, stigma theories were reviewed, and the multidimensional concept of public stigma was extracted. Second, items will be pooled by reviewing previous studies on existing public stigma scales. Third, data will be collected through in-depth, semi-structured interviews. This phase aims to explore the constructs and components of public stigma toward women undergoing infertility treatment in Japan.

#### 2.1.1 Identification of the multidimensional concept of public stigma

Stigma is a multidimensional concept (17). Corrigan et al. (18) identified three components of mental health-related public stigma using social attribution theory. First, *stereotyping (cognitions)*, pertains to beliefs of laypeople regarding the members of the minority group. Second, *prejudice (emotional reactions)*, refers to the endorsement of negative stereotypes by laypeople, resulting in negative emotional reactions. Third, *discrimination (behavioral responses)*, highlights the aspects of harm based on prejudice against the minority group including social distance and interpersonal avoidance. In addition, a fourth component, *attitudes toward cultural and institutional context*, contributes to deviance (19).

#### 2.1.2 Data collection through literature review

The item pool will be generated through a review of literature on existing scales measuring various illness-related stigmas in laypeople and relevant items. Based on systematic reviews and previous studies (17, 20, 21), illness-related public stigma scales were identified. Supplementary material 1 indicates the studies to be included in the item pool. A total of 722 items from 32 public stigma scale development studies on mental health, HIV/AIDS, leprosy, stroke, cancer, COVID-19, dementia, pregnant smoker, epilepsy, and chronic diseases will be included in the item pool, along with 86 items from three studies that developed scales to measure perceptions of infertility-related stigma, resulting in 808 items from 35 studies. All items extracted from the literature review will be translated into Japanese. Duplicate items and those not easily adaptable to infertility-related situations will be removed. The remaining items will be organized into four categories—cognitions, emotional reactions, behavioral responses, and attitudes toward the cultural and institutional context —based on the identification of the multidimensional concept of public stigma.

#### 2.1.3 Data generation through interviews

To construct more applicable items, we will interview laypeople using a semi-structured open-ended method. An interview guide consisting of open-ended questions will be developed by an expert panel (*n* = 7; four doctors, one nurse, and two researchers) to identify the components of infertility-related public stigma: cognitions, emotional reactions, behavioral responses, and attitudes toward cultural and institutional contexts. The potential interview outlines include: (1) What do you know about women undergoing infertility treatment? How do you feel about them? (2) Have you ever interacted with a woman undergoing infertility treatment? How were they? How did you feel during these experiences? (3) What do you think are the causes of infertility? (4) What actions do you take toward women undergoing infertility treatment? Do you think they should be treated differently from others? (5) How is infertility regarded in Japanese culture?

Participants will be selected using a purposive sampling with maximum variation in sex, age, occupation, place of residence, medical history, and experience of contact with women with primary or secondary infertility (22). Interviews will be transcribed by RY. Because this phase follows a qualitative research design, the sample size does not need to be determined in advance (22), and participants will be recruited until data saturation is reached (i.e., when no new themes emerge) (22).

#### 2.1.4 Participant characteristics and research setting

We will not use convenience or snowball sampling to recruit participants from across Japan. Laypeople over 20 years enrolled in a Japanese research firm’s database will be invited via email to participate in an interview. After logging in, potential participants will select the study from a list of surveys. If they wish to participate, they will proceed to screening based on eligibility criteria and purposive sampling. The eligible participants will then be sampled to maximize diversity. The inclusion criteria are as follows: (1) individuals over 20 years of age and (2) those who consent to participate in this study. The exclusion criteria are as follows: (1) currently undergoing or having undergone infertility treatment, (2) couples currently undergoing or having undergone infertility testing, (3) couples diagnosed with male factor infertility, (4) individuals with a background or experience in healthcare, and (5) individuals diagnosed with dementia. Eligible participants will be selected using maximum variation sampling based on sex, age, occupation, place of residence, medical history, and contact with women undergoing infertility treatment. Sampled participants will receive an email with the scheduled interview date, time, Zoom meeting ID, and password. Interviews will be conducted individually by RY via Zoom. The older adult may not have access to the Internet. If older participants cannot be recruited through the Japanese research firm’s database, they will be recruited from users of home-visit care providers. When interviewing older adults, obtaining accurate responses is especially important to minimize memory bias. To achieve this, the following strategies will be implemented (23, 24). (1) Minimizing the impact of the interviewer: efforts will be made to reduce the interviewer’s impact. For example, open-ended questions will be asked after closed-ended ones to encourage further elaboration. (2) Using clear, unambiguous and plain language: the interviewer will use expressions that are less ambiguous and less likely to cause misunderstandings. This includes avoiding double-barreled questions, maintaining clear sentence structures, and refraining from using technical terms. (3) Preparing paraphrases in advance: before conducting interviews, alternative phrasing or synonymous expressions will be prepared to enhance clarity and comprehension. (4) Observing facial expressions: during interviews, attention will be paid to participants’ facial expressions to assess their understanding of the questions. (5) Using repetition, summarization, and paraphrasing techniques: these methods will be used to clarify key points and ensure accurate responses. (6) Increasing sample size when necessary: in some cases, the number of participants may be increased.

#### 2.1.5 Data analysis

Qualitative-descriptive methods and inductive thematic analysis will be used in this phase (25–30). A qualitative-descriptive study is a comprehensive summary of events in everyday terms (29). That is, this study will have less abstract findings than other qualitative research methods because they are described in everyday terms (29). Additionally, the qualitative-descriptive research method is suitable for examining people’s reactions (thoughts, feelings, and attitudes) to an event (26). Because the items of the scale use everyday terms and the components of stigma reflect people’s responses, a qualitative-descriptive research method was chosen. We will also use thematic analysis in this phase because it is often used in qualitative-descriptive studies (28). Thematic analysis is the systematic process of identifying patterns in qualitative data (30).

To ensure the rigor of this qualitative-descriptive research, the following techniques will be used to increase the credibility, transferability, dependability, and confirmability of the study (22): purposive sampling, maximum variation sampling, thick description, data (source) triangulation, researcher triangulation, audit trail, and peer review. All data will be analyzed using Microsoft Excel version 2,407.

### 2.2 Development of the initial items

The results of the literature review and qualitative-descriptive study will be used to create an item pool, which will be reviewed by an expert panel (*n* = 7; four doctors, one nurse, and two researchers) to create the initial items of the Infertility Public Stigma Scale for Japanese women.

#### 2.2.1 Content validity through expert panel

Content validity will be assessed according to the guidelines and previous research (31–35). In general, expert panels assessing the content validity of a scale consist of 3 to 20 panel members (31). Accordingly, the expert panel of this phrase comprises four doctors, one nurse, two researchers, and two women who had undergone infertility treatment (*n* = 9). They will engage in a consensus group discussion on grammar conformity, appropriate word choice, word order in each item, and the scoring method to be employed (36). The items will be revised accordingly. The expert panel members will respond to a questionnaire about the essentiality, relevance, clarity of each item, and comprehensiveness of each dimension and the entire instrument. The content validity ratio, item-level content validity index (I-CVI) for relevance and clarity, modified kappa, and scale-level content validity index/average and universal agreement will allow assessment of consensus. Based on these results, the items will be modified or deleted.

#### 2.2.2 Content validity through cognitive interviews

Following the empirical literature (37, 38), this scale will be tested in two or three rounds with seven laypeople, selected using convenience and snowball sampling. This is because the ideal sample size for cognitive interviews assessing content validity ranges from 5 to 15 participants, conducted in two or three rounds (37). Interviews will be conducted using a guide based on previous studies (39, 40) and carried out via the Zoom application. The participants will be interviewed on (1) their general impression of the scale; (2) the comprehensibility of the items; (3) relevance to laypeople’s cognitive, emotional, and behavioral reactions; (4) the comprehensiveness of the items; (5) suggested revisions; and (6) appropriateness of the response options. Accordingly, the scale or items will be modified or deleted.

### 2.3 The quantitative phase: scale validation

The quantitative phase will examine the reliability and validity of the Infertility Public Stigma Scale for Japanese women, including structural validity, internal consistency, inter-rater reliability, and known-groups, convergent, and discriminant validity.

#### 2.3.1 Participants and recruitment procedure

A cross-sectional study based on an online survey of laypeople will be conducted to examine the scale’s reliability and validity. The participants will be recruited from a Japanese research firm’s database of individuals over 20 years old. Potential participants will be invited via email and screened to determine eligibility if they wish to participate. Eligible individuals will respond to a questionnaire on the company’s website. The inclusion criteria will be as follows: (1) individuals over 20 years old and (2) those who have consented to participate in this study. Exclusion criteria will be as follows: (1) individuals currently undergoing or who have undergone infertility treatment, (2) couples currently undergoing or those who have undergone infertility testing, (3) couples diagnosed with male factor infertility, (4) individuals with a background or experience in healthcare, and (5) individuals diagnosed with dementia.

Convenience sampling will be used to recruit both male and female participants of diverse ages in order to represent the Japanese population. Older adult may not have access to the Internet. If it is not possible to recruit elderful individuals from the databases of the Japanese research firm, they will be recruited from users of home-visit care providers. To examine the reliability and validity of the scale, 10–15 participants are needed per candidate item (41). Therefore, the target sample size will be calculated by multiplying the number of candidate items by 10–15.

#### 2.3.2 Data collection

Data will be collected on sociodemographics, health status, contact with women undergoing infertility treatment, and fertility knowledge.

Sociodemographic factors and health status will include age, sex, education, occupation, annual household income, residential area, medical history, and contact with women having primary or secondary infertility.

In addition, we will collect data using the Japanese version of the Cardiff Fertility Knowledge Scale (CFKS-J) (42), developed by Maeda et al. The original version was developed by Bunting et al. (43). The CFKS-J consists of 13 items that measure risks for reduced fertility, misconceptions about fertility, and basic facts about infertility, with each item rated on a 3-point scale.

#### 2.3.3 Data analysis

The data will be analyzed using the R version 4.4.1 for Windows. Statistical significance will be set at *p* < 0.05. Descriptive statistics of means, percentages, and standard deviations (SD) will be calculated for sociodemographic factors and health status.

##### 2.3.3.1 Item exclusion criteria

We will remove items that show either ceiling or floor effects. We will also remove the items if item-total and item-remainder correlations are below 0.4 or above 0.85. Items with factor loadings less than 0.4 in the exploratory factor analysis will be considered for deletion.

##### 2.3.3.2 Examination of the structural validity

Exploratory factor analysis will be conducted to examine the structural validity of the Infertility Public Stigma Scale for Japanese women. After confirming the factor numbers using a scree plot, we will check whether the data are multivariate normally distributed, using Mardia’s kurtosis test, and determine the extraction method accordingly (44). In addition, a confirmatory factor analysis will be conducted based on the results of the exploratory factor analysis. The extraction method will be determined based on whether the data follows a multivariate normal distribution (45). A chi-square statistic / degrees of freedom less than or equal to 3 will be considered appropriate (46). In addition, a good fit will be considered if (1) the goodness of fit index, comparative fit index, Tucker-Lewis index, adjusted goodness of fit index, and normed fit index are greater than or equal to 0.95 and (2) the root mean square error of approximation and standardized root mean square residual are less than or equal to 0.05 (47–49).

##### 2.3.3.3 Examination of reliability

To examine internal consistency, Cronbach’s alpha coefficients, as well as item-total and item-remainder correlations, will be calculated. A Cronbach’s alpha coefficients greater than 0.7 will be considered optimal (50). To assess test–retest reliability, we will administer the Infertility Public Stigma Scale to more than 50% of the participants 3 days after the initial survey (33). If the data follow a normal distribution, we will calculate an intraclass correlation coefficient based on the Infertility Public Stigma Scale scores at baseline and after 3 days (51).

##### 2.3.3.4 Examination of the known-groups, convergent, and discriminant validity

A known-groups comparison will be conducted using a t test if the data follow a normal distribution. We assume that the mean scores for individuals aged 60 and older will be higher than that for those who are younger (3).

To examine convergent validity, Pearson’s correlation coefficient will be calculated between the Infertility Public Stigma Scale and the CFKS-J. In the context of mental illness, stereotyping arises when responsibility for the illness is attributed to the individual and they are blamed (18). Therefore, we expect a significant positive correlation coefficient between infertility public stigma and the CFKS-J.

Convergent and discriminant validity will be assessed using a multitrait-scaling analysis. Convergent validity is sustained if modest correlation of 0.4 or greater is observed between an item and its own scale (51). Discriminant validity is sustained when the correlation between an item and its own scale is higher than the item’s correlation with other scales (51). Scaling errors and successes will then be calculated (51).

## 3 Discussion

This protocol article described a plan for the development of the Infertility Public Stigma Scale for Japanese women and the examination of its reliability and validity. This study will describe the concept and components of public stigma toward infertile patients and develop the Infertility Public Stigma Scale for Japanese women. The results of this study are expected to accelerate our understanding of the situation faced by women undergoing infertility treatment and contribute to the development of programs aimed at reducing public stigma toward them. The results of this study (scale development) are expected to be academically significant, as they can be used to measure the effectiveness of programs aimed at reducing public stigma of infertility. Furthermore, the need to reduce public stigma regarding infertility may be revealed by determining its extent.

### 3.1 Limitations

This study has some limitations. Participants in the interviews conducted during the qualitative phase of this study will be recruited from online research firm monitors. Participant selection and sampling biases must be considered when interpreting the findings of this study. However, the ability to collect data from both men and women across Japan allows for sampling with maximum variation. Second, the nature of the participant recruitment process for the assessment of the scale’s reliability and validity may introduce participant selection and sampling bias. Despite these limitations, this study will be the first to examine the reliability and validity of the Infertility Public Stigma Scale for Japanese women.
