# Supplementary material for: Development and psychometric evaluation of the Infertility Public Stigma Scale for Japanese women: protocol for an exploratory sequential mixed method study of the Japanese population
Source: Front Public Health. 2025 May 21;13:1504842. doi: 10.3389/fpubh.2025.1504842 (PMC12133471; doi:10.3389/fpubh.2025.1504842)
Supplement: Supplementary file 1 [file Table_1.docx]

Supplementary Material

# Supplementary Table 1: Studies to be included in the item pool through literature reviews

|  | Source | Illness | Country | Item extracted |
| --- | --- | --- | --- | --- |
| Public stigma measures [1-3] | |  |  |  |
| [4] | Taylor & Dear (1981) | Mental Illness | Canada | 40 |
| [5] | Burra et al. (1982) | Mental Illness | Canada | 30 |
| [6] | Link (1987) | Mental Illness | USA | 12 |
| [7] | Link et al. (1989) | Mental Illness | USA | 15 |
| [8] | Penn et al. (1994) | Mental Illness | USA | 37 |
| [9] | Corrigan et al. (2002) | Mental Illness | USA | 20 |
| [10] | Corrigan et al. (2003) | Mental Illness | USA | 28 |
| [11] | Schulze et al. (2003) | Mental Illness | Germany | 19 |
| [12] | Angermeyer & Matschinger (2004) | Mental Illness | Germany | 27 |
| [13] | Watson, Miller & Lyons (2005) | Mental Illness | USA | 21 |
| [14] | Kassam et al. (2010) | Mental Illness | Canada | 28 |
| [15] | O'Reilly et al. (2010) | Mental Illness | Australia | 26 |
| [16] | Kassam et al. (2012) | Mental Illness | Canada | 20 |
| [17] | Ferchaud et al. (2020) | Mental Illness | USA | 15 |
| [18] | Froman, Owen & Daisy (1992) | HIV/AIDS | USA | 21 |
| [19] | Green (1995) | HIV/AIDS | Scotland | 15 |
| [20] | O'Hea et al. (2001) | HIV/AIDS | USA | 27 |
| [21] | Kalichman et al. (2005) | HIV/AIDS | South Africa | 9 |
| [22] | Visser et al. (2008) | HIV/AIDS | South Africa | 34 |
| [23] | Zelaya et al. (2008) | HIV/AIDS | USA | 24 |
| [24] | Bresnahan & Zhuang (2010) | HIV/AIDS | USA | 27 |
| [25] | Lau & Chan (2010) | HIV/AIDS | China | 19 |
| [26] | Adrien et al. (2013) | HIV/AIDS | Canada | 16 |
| [27] | Beaulieu et al. (2014) | HIV/AIDS | Canada | 42 |
| [28] | Peters et al. (2014) | Leprosy | Netherlands | 22 |
| [29] | Wan et al. (2023) | Stroke | China | 33 |
| [3] | Marlow & Wardle (2014) | Cancer | UK | 26 |
| [30] | Nochaiwong et al. (2021) | COVID-19 | Thailand | 10 |
| [31] | Kim et al. (2022) | Dementia | Australia | 16 |
| [32] | Loyal et al. (2022) | Pregnant Smoker | France | 26 |
| [33] | Austin et al. (2002) | Epilepsy | USA | 7 |
| [34] | Fekih-Romdhane et al. (2023) | Chronic diseases | Tunisia | 10 |
| Infertility stigma measures | |  |  |  |
| [35] | Yokota et al. (2022) | Infertility | Japan | 27 |
| [36] | Taebi et al. (2022) | Infertility | Iran | 20 |
| Negative social interactions | |  |  |  |
| [37] | Akizuki (2020) | Infertility | Japan | 39 |
|  | **Total** |  |  | 808 |

1. Kaushik A, Kostaki E, Kyriakopoulos M. The stigma of mental illness in children and adolescents: A systematic review. Psychiatry Res. 2016;243:469-94.

2. Rodríguez-Rivas ME, Cangas AJ, Cariola LA, Varela JJ, Valdebenito S. Innovative Technology-Based Interventions to Reduce Stigma Toward People With Mental Illness: Systematic Review and Meta-analysis. JMIR Serious Games. 2022;10(2):e35099.

3. Marlow LA, Wardle J. Development of a scale to assess cancer stigma in the non-patient population. BMC Cancer. 2014;14:285.

4. Taylor SM, Dear MJ. Scaling community attitudes toward the mentally ill. Schizophr Bull. 1981;7(2):225-40.

5. Burra P, Kalin R, Leichner P, Waldron JJ, Handforth JR, Jarrett FJ, et al. The ATP 30-a scale for measuring medical students' attitudes to psychiatry. Med Educ. 1982;16(1):31-8.

6. Link BG. Understanding labeling effects in the area of mental disorders: An assessment of the effects of expectations of rejection. American Sociological Review. 1987;52(1):96-112.

7. Link BG, Cullen FT, Frank J, Wozniak JF. The social rejection of former mental patients: Understanding why labels matter. American Journal of Sociology. 1987;92(6):1461-500.

8. Penn DL, Guynan K, Daily T, Spaulding WD, Garbin CP, Sullivan M. Dispelling the stigma of schizophrenia: what sort of information is best? Schizophr Bull. 1994;20(3):567-78.

9. Corrigan PW, Rowan D, Green A, Lundin R, River P, Uphoff-Wasowski K, et al. Challenging two mental illness stigmas: personal responsibility and dangerousness. Schizophr Bull. 2002;28(2):293-309.

10. Corrigan P, Markowitz FE, Watson A, Rowan D, Kubiak MA. An attribution model of public discrimination towards persons with mental illness. J Health Soc Behav. 2003;44(2):162-79.

11. Schulze B, Richter-Werling M, Matschinger H, Angermeyer MC. Crazy? So what! Effects of a school project on students' attitudes towards people with schizophrenia. Acta Psychiatr Scand. 2003;107(2):142-50.

12. Angermeyer MC, Matschinger H. The stereotype of schizophrenia and its impact on discrimination against people with schizophrenia: results from a representative survey in Germany. Schizophr Bull. 2004;30(4):1049-61.

13. Watson AC, Miller FE, Lyons JS. Adolescent attitudes toward serious mental illness. J Nerv Ment Dis. 2005;193(11):769-72.

14. Kassam A, Glozier N, Leese M, Henderson C, Thornicroft G. Development and responsiveness of a scale to measure clinicians' attitudes to people with mental illness (medical student version). Acta Psychiatr Scand. 2010;122(2):153-61.

15. O'Reilly CL, Bell JS, Chen TF. Consumer-led mental health education for pharmacy students. Am J Pharm Educ. 2010;74(9):167.

16. Kassam A, Papish A, Modgill G, Patten S. The development and psychometric properties of a new scale to measure mental illness related stigma by health care providers: the Opening Minds Scale for Health Care Providers (OMS-HC). BMC Psychiatry. 2012;12:62.

17. Ferchaud A, Seibert J, Sellers N, Escobar Salazar N. Reducing Mental Health Stigma Through Identification With Video Game Avatars With Mental Illness. Front Psychol. 2020;11:2240.

18. Froman RD, Owen SV, Daisy C. Development of a measure of attitudes toward persons with AIDS. Image J Nurs Sch. 1992;24(2):149-52.

19. Green G. Attitudes towards people with HIV: are they as stigmatizing as people with HIV perceive them to be? Soc Sci Med. 1995;41(4):557-68.

20. O'Hea EL, Sytsma SE, Copeland A, Brantley PJ. The Attitudes Toward Women with HIV/AIDS Scale (ATWAS): development and validation. AIDS Educ Prev. 2001;13(2):120-30.

21. Kalichman SC, Simbayi LC, Jooste S, Toefy Y, Cain D, Cherry C, et al. Development of a brief scale to measure AIDS-related stigma in South Africa. AIDS Behav. 2005;9(2):135-43.

22. Visser MJ, Kershaw T, Makin JD, Forsyth BW. Development of parallel scales to measure HIV-related stigma. AIDS Behav. 2008;12(5):759-71.

23. Zelaya CE, Sivaram S, Johnson SC, Srikrishnan AK, Solomon S, Celentano DD. HIV/AIDS stigma: reliability and validity of a new measurement instrument in Chennai, India. AIDS Behav. 2008;12(5):781-8.

24. Bresnahan M, Jie Z. Exploration and validation of the dimensions of stigma. J Health Psychol. 2011;16(3):421-9.

25. Lau JT, Tsui HY, Chan K. Reducing discriminatory attitudes toward people living with HIV/AIDS (PLWHA) in Hong Kong: an intervention study using an integrated knowledge-based PLWHA participation and cognitive approach. AIDS Care. 2005;17(1):85-101.

26. Adrien A, Beaulieu M, Leaune V, Perron M, Dassa C. Trends in attitudes toward people living with HIV, homophobia, and HIV transmission knowledge in Quebec, Canada (1996, 2002, and 2010). AIDS Care. 2013;25(1):55-65.

27. Beaulieu M, Adrien A, Potvin L, Dassa C. Stigmatizing attitudes towards people living with HIV/AIDS: validation of a measurement scale. BMC Public Health. 2014;14:1246.

28. Peters RM, Dadun, Van Brakel WH, Zweekhorst MB, Damayanti R, Bunders JF, et al. The cultural validation of two scales to assess social stigma in leprosy. PLoS Negl Trop Dis. 2014;8(11):e3274.

29. Wan M, Tan Y, Huang Y, Zhang Q, Qin F, Sun X, et al. Development and psychometric evaluation of public stigma of stroke scale (PSSS). Sci Rep. 2023;13(1):545.

30. Nochaiwong S, Ruengorn C, Awiphan R, Kanjanarat P, Ruanta Y, Phosuya C, et al. COVID-19 Public Stigma Scale (COVID-PSS): development, validation, psychometric analysis and interpretation. BMJ Open. 2021;11(11):e048241.

31. Kim S, Eccleston C, Klekociuk S, Cook PS, Doherty K. Development and psychometric evaluation of the Dementia Public Stigma Scale. Int J Geriatr Psychiatry. 2022;37(2).

32. Loyal D, Sutter ALMDP, Auriacombe M, Serre F, Rascle N. The Pregnant Smoker Stigma Scale - Public Stigma (P3S-PS): development and validation in general French population. Women Health. 2022;62(2):157-67.

33. Austin JK, Shafer PO, Deering JB. Epilepsy familiarity, knowledge, and perceptions of stigma: report from a survey of adolescents in the general population. Epilepsy Behav. 2002;3(4):368-75.

34. Fekih-Romdhane F, Obeid S, Chidiac G, Dabbous M, Malaeb D, Hallit R, et al. Measuring public attitudes towards people living with chronic diseases in Arabic-speaking populations: adaptation and development of the Social Stigma Scale of Chronic Diseases (SSS-CD). BMC Public Health. 2023;23(1):1375.

35. Yokota R, Okada H, Okuhara T, Goto E, Furukawa E, Shirabe R, et al. Development of the Japanese Version of the Infertility Stigma Scale: Examination of Its Reliability and Validity. Healthcare (Basel). 2022;10(3).

36. Taebi M, Kariman N, Montazeri A, Alavi Majd H, Jahangirifar M. Development and psychometric properties of Female Infertility Stigma Instrument (ISI-F): A sequential mixed method study. BMC Womens Health. 2022;22(1):557.

37. Akizuki Y, Kai I. Source-specific effects of negative social interactions and positive social interactions on depression in infertile Japanese women. *J Jp Soc Psychosom Obstet Gynecol*. 2020:103-14.
